# Efficacy of *Ficus carica* leaf extract on morphological and molecular behavior of mice germ stem cells

**DOI:** 10.1590/1984-3143-AR2022-0036

**Published:** 2022-08-19

**Authors:** Zohreh Makoolati, Hasan Bahrami, Zahra Zamanzadeh, Maryam Mahaldashtian, Alireza Moulazadeh, Lida Ebrahimi, Majid Naghdi

**Affiliations:** 1 Department of Anatomical Sciences, Faculty of Medicine, Fasa University of Medical Sciences, Fasa, Iran; 2 Department of Genetic, Basic Sciences Faculty, Nour Danesh Institute of higher Education, Isfahan, Iran; 3 Department of Biotechnology, Faculty of Biological Sciences and Technology, Shahid Ashrafi Esfahani University, Isfahan, Iran; 4 Non Communicable Disease Research Center, Fasa University of Medical Sciences, Fasa, Iran; 5 Department of Tissue Engineering, School of Advanced Technologies, Fasa University of Medical Sciences, Fasa, Iran

**Keywords:** spermatogonial stem cell, proliferation, Ficus carica, cytotoxicity, viability

## Abstract

Infertility is one of the most prevalent health disorders in reproductive-age males and females. *Ficus carica* (Fc), an herbal plant, has been used traditionally for the treatment of different diseases such as infertility especially in Iranian folk medicine. This study examined the effects of Fc leaf extract on the proliferation of mice spermatogonial stem cells (SSCs). Phenolic, flavonoid content, major polyphenolic compounds and antioxidant activity of the extract was evaluated respectively by Folin-Ciocateu, aluminum chloride, HPLC and the FRAP and DPPH methods. Testicular cells of neonate mice were extracted and their identity was confirmed using cytokeratin for Sertoli and Oct-4, CDHI and PLZF for SSCs. Effects of Fc (0.0875, 0.175, 0.35, 0.71 and 1.42 mg/ml) was evaluated at third, 7th, 9th and 14th days of culture by colony assay. The expression of the Mvh, GFRα1 and Oct-4 genes and the viability and proliferation of cultured cells was assessed at the end of the culture period. The extract has a rich phenolic and flavonoid content such as Rutin, Psoralen, Bergapten and Caffeoylmalic acid using HPLC analysis. It also had a potent reducing and radical scavenging activity. Morphology of colonies was similar in all groups. Higher viability, proliferation, colony number and diameter of SSCs was seen in the presence of Fc leaf extract in a dose-dependent manner so that higher number and diameter of colonies were observed in two higher doses of 0.71 and 1.42 mg/ml, separately for each time point relative to other groups. The Mvh, Oct-4 and GFRα1 genes expression had no significant differences between groups. It seems that Fc leaf extract not only had no any cytotoxic effects on the viability and proliferation of SSCs but also support their stemness state. So, this culture system can be employed for enrichment of germ stem cells for use in clinical applications.

## Introduction

Infertility is one of the most serious social problems of reproductive-age males and females (Radford et al., 1999). Male partner factors are responsible for about half of all cases of infertility (Miyamoto et al., 2012). One of the major reasons of infertility in males is oligospermia or azoospermia (Radford et al., 1999). Spermatogonial stem cells (SSCs) of the testis are able to self-renew and produce differentiated sperms (Kanatsu-Shinohara et al., 2005). This regenerative potential of SSCs make this cell a key candidate for infertility treatment (Brinster and Nagano, 1998). Thus, it is essential to use methods for the separation and long-term cultivation of this cell (Zhang et al., 2011). Owing to the low number of germ stem cells, isolation of SSCs is difficult (Morena et al., 1996). Also, it has been reported that apoptosis is the leading cause of SSCs decrease during the first week of culture (Aponte et al., 2006). Reactive oxygen species (ROS) have been identified as major contributing factors for Apoptosis (Naskar et al., 2010), which increase in the culture media compared to *in vivo* condition (Luvoni et al., 1996). Several protocols of *in vitro* SSCs enrichment had been developed up to now. One of the most significant current attention of *in vitro* SSCs enrichment is the use of antioxidants to break the oxidative chain reaction (Naskar et al., 2010). In recent years, there have been increasing amounts of literature on herbal medicines as the preferred choice for infertility treatment due to the presence of antioxidants in plants (Safarnavadeh and Rastegarpanah, 2011; Mahaldashtian et al., 2015, 2016; Naghdi et al., 2016).

Fig (*Ficus carica L*.), from the Moraceae family, is one of the plants mainly used for this purpose (Trifunski and Ardelean, 2012; Naghdi et al., 2016). Antioxidant compounds are an important content in the leaf, pulp and skin of fig, with a higher amount in leaf, which play a key role in the elimination of free radicals (Oliveira et al., 2009; Aghel et al., 2011; Oh et al., 2011; Samsulrizal et al., 2011). Fig tree leaves has been used as a folk remedy for curing different disorders in Iranian traditional medicine described by Avicenna (Hashemi and Abediankenari, 2013). In 2011, Samsulrizal et al. (2011) demonstrated that oral intake of *Ficus deltoidea* leaf extract led to the decrease of sperm abnormality and improved sperm count and motility as well as the level of testosterone of diabetic rats. Studies such as one conducted by Naghdi et al. (2016) have shown that the leaf extract of Fc enhanced non-progressive motility and count of sperms, as well as the gonadosomatic index in testes of formaldehyde treated mice. Also, after administration of FC leaf extract, spermatogenic arrest was rarely seen in seminiferous tubule of testis exposed to formaldehyde. Based on a compilation by Haredy et al., FC ameliorated Lithium carbonate -induced reduction in reproductive performance of male rats through its antioxidant and antiapoptotic properties (Haredy et al., 2017). A recent study also reported the profertility effect of FC leaf extract on streptozotocin-induced diabetic male rats. Abu Bakar et al. (2020) found that FC has antihyperglycemic potential, and improves sperm quality parameters and increases the total protein expression especially those related to the fertility (Abu Bakar et al., 2020).

Studies conducted so far, have tended to focus on *in vivo* effects of herbs in infertility rather than *in vitro* assessment. This study critically examined the *in vitro* efficacy of FC leaf extract on the colonization of SSCs co-culture with Sertoli cells.

## Methods

### Reagents

Fetal bovine serum (FBS), Trypsin, cytokeratin, hyaloronidase, normal goat serum, and collagenase were purchased from Sigma- Aldrich (St Louis, MO, USA). PLZF (promyelocytic leukemia zinc finger), mouse CDH1 (cadherin-1), mouse Oct-4, and secondary FITC goat anti-mouse and goat anti-rabbit antibodies were supplied from R&D Systems, Calbiochem (San Diego, CA, USA), Chemicon (UK), and Raybiotech (Tehran, Iran), respectively. Triton X-100 was purchased from ICN Biomedicals and Dulbecco’s Modified Eagle Medium (DMEM) was the product of Gibco-BRL, (Grand Island,NY, USA).

### Ethical statements

NMRI (National Medical Research Institute) mice were purchased from Shiraz Medical University and kept at the Fasa University of Medical Science’s animal house. Animals were subjected to the standard conditions of maintenance. The Ethics Committee of the College of Fasa University of Medical Sciences approved the study (the approval number of the Research Ethical Committee is 93130). All trials were done in agreement with relevant guidelines and protocols.

### Fig leaf processing

Since *Ficus carica* is deciduous, the leaves were prepared from Shiraz Area Plant garden, South of Iran, from the end of spring to the beginning of autumn. The leaves were validated (voucher specimen No: 100-2) and preserved in the Herbarium of the Fasa University. About 10 g of the power of blended leaves was liquefied in 125mL of alcohol 80% and shaked using a magnetic shaker for 3 days. Following filtration (twice), lyophilization in 55–60°c oven and drying, it was resolved in normal saline.

### Measurement of phenolic content

To measure the phenolic content of the *Ficus carica* leaf extract, the Folin-Ciocalteu assay was used. 500 μl of 10% Folin-Ciocalteu reagent (10% v/v) was added to 100 μl of the Fc extract at a concentration of 1 mg/ml and incubated for 5 minutes at room temperature and darkness. Then 400 μl of sodium carbonate (7.5% w/v) was added to the sample and the resulting solution was kept at room temperature and darkness for 60 minutes. Finally, the absorbance of the samples was measured by a Synergy HTX multi-mode reader at 765 nm wavelength. Gallic acid was also used as the standard and the phenol content of the Fc extracts was reported in microgram Gallic Acid Equivalent (GAE) per milligram of dry weight (µg GAE/mg) (Dzah et al., 2020a, b). Phenolic content measurement of the Fc extract was repeated three times.

### Evaluation of flavonoid content

To measure the flavonoid content of the Fc leaf extract, the aluminum-chloride assay was used. 50 μl of aluminum chloride (10% w/v) and 50 μl of sodium nitrite (5% w/v) were added to 200 μl of the Fc extract at a concentration of 1 mg/ml. The resulting solution was incubated for 5 minutes at room temperature and darkness. Subsequently, 700 μl of sodium hydroxide (4% w/v) was added. The total volume of the solution was equal to 1 ml and incubated for 15 minutes at room temperature and darkness. Finally, the absorption of the solution was read at 510 nm using a Synergy HTX multi-mode reader. Quercetin was used as the standard and the flavonoid content of the Fc extract was reported in micrograms Quercetin Equivalent (QE) per milligram of dry weight (µg QE/mg) (Aryal et al., 2019; Shi et al., 2018). Flavonoid content measurement of the Fc extract was repeated three times.

### HPLC analysis of major phenolic and flavonoid compounds

A waters liquid chromatography apparatus consisting of a Separations module: waters 2695 (USA) and a PDA Detector waters 996 (USA) was used for the HPLC analysis. Data acquisition and integration was performed with Millennium32 software. The chromatographic assay was performed on a 15 cm×4.6 mm with pre-column, Eurospher 100-5 C_18_ analytical column provided by waters (sunfire) reversed phase matrix (3.5 μm) (Waters) and elution was carried out in a gradient system with methanol as the organic phase (solvent A) and distilled water (solvent B) with the flow-rate of 1 ml/ min. Peaks were monitored at 195-400 nm wavelength. Injection volume was 20 µL and the temperature was maintained at 25°C.

### Evaluation of antioxidant activity

FRAP (Ferric Reducing Antioxidant Power) and DPPH (2,2-diphenyl-1-picrylhydrazyl) assay were used to evaluate the antioxidant activity of the Fc leaf extract. The FRAP assay is used to evaluate the monovalent antioxidants that reduce Fe^3+^ ions to Fe^2+^; Whereas DPPH assay evaluate the total antioxidant activity of the Fc extract by evaluating the reduction process of stable nitrogen radicals (DPPH radicals) (Feghhi-Najafabadi et al., 2019; Sethi et al., 2020).

### Evaluation of the monovalent reducing power

In the FRAP assay, the antioxidant activity of the Fc extract to reduce Fe^3+^ ions (contained in the Fe-TPTZ complex) and converting to Fe^2+^ ions is measured. According to previous studies, 1.5 ml of the FRAP working solution was poured into a test tube and 50 μl of the sample or standard was added and mixed. After 10 minutes of incubation at 37 ° C, the absorption of the sample was read at 593 nm. FeSO4 solution (serial dilution of 1mM concentration) was also used as the standard and the antioxidant activity of the Fc extract was reported in μmol Fe^2+^/gr (Wang et al., 2016).

### Determination of total radical scavenging activity

In the DPPH clearance assay, the total radical scavenging activity of the Fc extract is determined.

DPPH radical has a purple color that turns yellow after reduction by antioxidants and its colorlessness indicates the antioxidant activity of the extract. Different concentration of the extract (50, 100, 200, 500, 1000, 2000 and 5000 µg/ml) was prepared. 40 μl of the extract was added to 160 μl of 0.3 mM DPPH, mixed and incubated for 30 minutes at room temperature and darkness. Finally, the absorption of the sample was measured in 517 nm by the Synergy HTX multi-mode reader. The antioxidant activity of Ascorbic acid was also measured as a powerful antioxidant compound. The control group was the Ethanol 70% (v/v) that used in dilution of the extract. The percentage of the antioxidant activity was calculated with the following formula (Sridhar and Charles, 2019): Antioxidant power = [(Optical absorption of the control group - Optical absorption of the experimental group) / Optical absorption of the control group] × 100.

### Isolation of testicular cells

Six to ten day old mice were sacrificed and their testes were digested by two step enzymatic digestion method (18) using an enzymatic digestion solution (0.5 mg/ ml hyaloronidase, trypsin and collagenase). In the first digestion step, tunica albogina was removed and testis tissue was fragmented in DMEM enclosing enzymatic solution in order to eliminate interstitial fibroblasts and endothelial cells. The next step was done as the same for the separation of germ cells and Sertoli cells from seminiferous tubules.

### Identity approval of extracted cells

The extracted cells were fixed for 20 min at the room temperature (RT) by paraformaldehyde (4%). The next steps were as the following: washing with PBS, incubation at RT for half an hour in HCl (2 N) and washing with borate buffer. Ten percent normal goat serum was used to block the nonspecific antibody reaction and the cells were treated using 0.3% triton X-100. All samples except negative control slides were kept in diluted cytokeratin (1/100), mouse Oct-4, CDH1 and PLZF antibodies for 60 min at 37 °C. After washing with PBS, secondary FITC goat anti-rabbit antibody (1/10 diluted) against PLZF and Oct-4 and goat anti-mouse antibody against CDH1 and cytokeratin for 120 min at RT were used. After that the counterstain with 5 µg/ml ethidium bromide was performed and the samples were washed and mounted with 90% glycerol.

### Cell culture

The equal primary number of extracted testicular cells was maintained under exponential growth in DMEM culture medium, supplemented with 10% FBS and medium was changed every 2 days. Cell cultures were maintained in an incubator containing 5% CO_2_.

### Extracting treatments

Cells were seeded in multi-well culture dishes to grow. Subsequently, the cultured cells were exposed to different concentrations (0.0875, 0.175, 0.35, 0.71 and 1.42 mg/ml) of FC leaf extract for 14 days.

### Cytotoxicity evaluation

Cytotoxicity effects of FC leaf extract on testicular cells was determined using viability percent and proliferation rate measurement. At the end of culture time, control and treated cells were trypsinized and stained with trypan blue (0.4%, Sigma). Counting was done using a hemocytometer. Proliferation rate was determined by counting the mean number of whole cells. Viability was defined as a percentage of live cells in a whole population.

### Colony assay

Colony assay was done between different days in all groups using the comparison of the mean number and diameter of SSCs colonies. This cluster assessment was performed during 2 weeks of culture on the third, 7th, 9th and 14th days of culture by an inverted microscope (Carl Zeiss; Germany)

#### RNA extraction, reverse transcription and real-time PCR

RNA was extracted from cultured cells using an RNXPlus™ according to manufacturer's guidelines. Reverse transcription was performed by RevertAid™ First strand cDNA synthesis kit and it was subsequently stored at −20 °C. For cDNA amplification, the StepOnePlus Real-Time PCR System (Applied Biosystems) was used. PCR was performed in capillaries with a PCR master mix (Fermentase, K0222) as specified by the manufacturer. The sequences of the Mvh, GFRα1, and Oct-4 oligonucleotide primers were F: 5' GCT CAA ACA GGG TCT GGG AAG3' and R:5' GGT TGA TCA GTT CTC GAG3' for Mvh, F:5' AAT TGT CTG CGT ATC TAC TGG 3' and R: 5' ACA TCT GAT ATG AAC GGG AC 3' for GFRα1, F:5' GAA CTA GCA TTG AGA ACC GT 3' and R:5' CAT ACT CGA ACC ACA TCC TTC 3' for Oct-4, and F:5' TCC CTG GAG AAG AGC TAC G 3' and R:5'GTA GTT TCG TGG ATG CCA CA 3' for β-actin. PCR was performed in the last bulk of 20 µL with 4 min at 94°C, followed by 40 cycles of 20 s at 94°C, 30 s at 60°C and 30 s at 72°C. Analysis of melt curve was done to confirm the amplification of the predicted fragments. The ratio of gene expression was determined by the comparative CT (cycle threshold) mode.

### Statistical analysis

All experiments were done in triplicate. Data were expressed as mean ± SD. Statistical differences among the groups were determined using one-way analysis of variance (ANOVA) followed by the Bonferroni and Tukey–Kramer multiple comparison test with the SPSS program (version 16.0) package. Statistically significance was accepted at *P* < 0.05. To determine the effect size, Partial Eta Squared (π_p_^2^) was used (Papp and Henninger, 2005).

## Results

### Phenolic and flavonoid content

**Figure 1:**
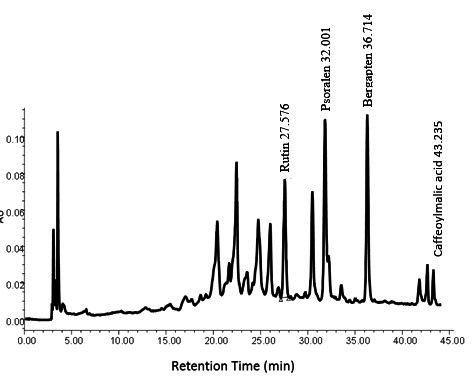
HPLC separation of phenolic and flavonoid content of Fc leaf extract.

The phenolic content of the Fc extract by the Folin Ciocalteu assay was 92.93 ± 4.38 µg GAE/mg and its flavonoid content by aluminum chloride assay was 181.01 ± 7.29 µg QE/mg. HPLC chromatogram of the Fc leaf extract is also shown in Figure 1. The chromatogram profile was consisted of 13 compounds; which Rutin, Psoralen, Bergapten and Caffeoylmalic acid were identified respectively by retention time of 27.576, 32.001, 36.714 and 43.235 min.

### Monovalent and total antioxidant activity

**Figure 2:**
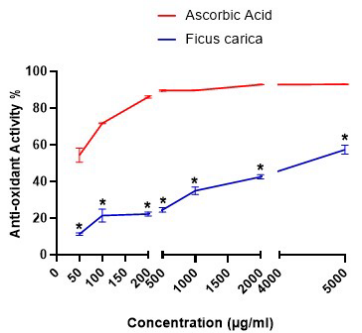
Total antioxidant activity of the Ficus carica leaf extract and Ascorbic Acid. The bullet (*) Indicates a statistically significant difference between the Fc extract and Ascorbic acid.

The monovalent antioxidant activity of the Fc extract was 472.66 ± 25.34 μmol Fe^2+^/g by FRAP assay. The total antioxidant activity (%) of the Fc extract was dose dependant and the IC50 value of the total antioxidant activity was 3459.64 µg/ml. According to the Table 1, the total antioxidant activity (%) on the 50 µg/ml concentration of the Fc extract was % 11.36 ± 0.74 and increased by 47.14% on 5000 µg/ml concentration of the Fc extract (% 58.50 ± 2.51). The total antioxidant activity of the extract at different concentrations was significantly (P<0.0001) less than Ascorbic acid (Figure 2). The IC50 value of the total antioxidant activity of Ascorbic acid was and 30.64 µg/ml.

**Table 1:** The total antioxidant activity (%) of the *Ficus carica* leaf extract and Ascorbic acid.

**CONC (µg/ml)**	**Ficus carica**		**Ascorbic Acid**		**P-value**
	**Mean ± SD**	**IC50**	**Mean ± SD**	**IC50**	
50	11.36 ± 0.74	3459.64	54.47 ± 3.88	30.64	< 0.0001
100	21.48 ± 3.51		72.02 ± 0.37		< 0.0001
200	22.35 ± 1.08		85.99 ± 0.68		< 0.0001
500	24.59 ± 1.28		89.63 ± 0.31		< 0.0001
1000	35.08 ± 2.02		89.77 ± 0.11		< 0.0001
2000	42.90 ± 1.08		92.95 ± 0.10		< 0.0001
5000	58.50 ± 2.51		93.06 ± 0.16		< 0.0001

CONC: concentration, IC50: Median inhibition concentration. P-value.

### Characterization of isolated testicular cells

**Figure 3:**
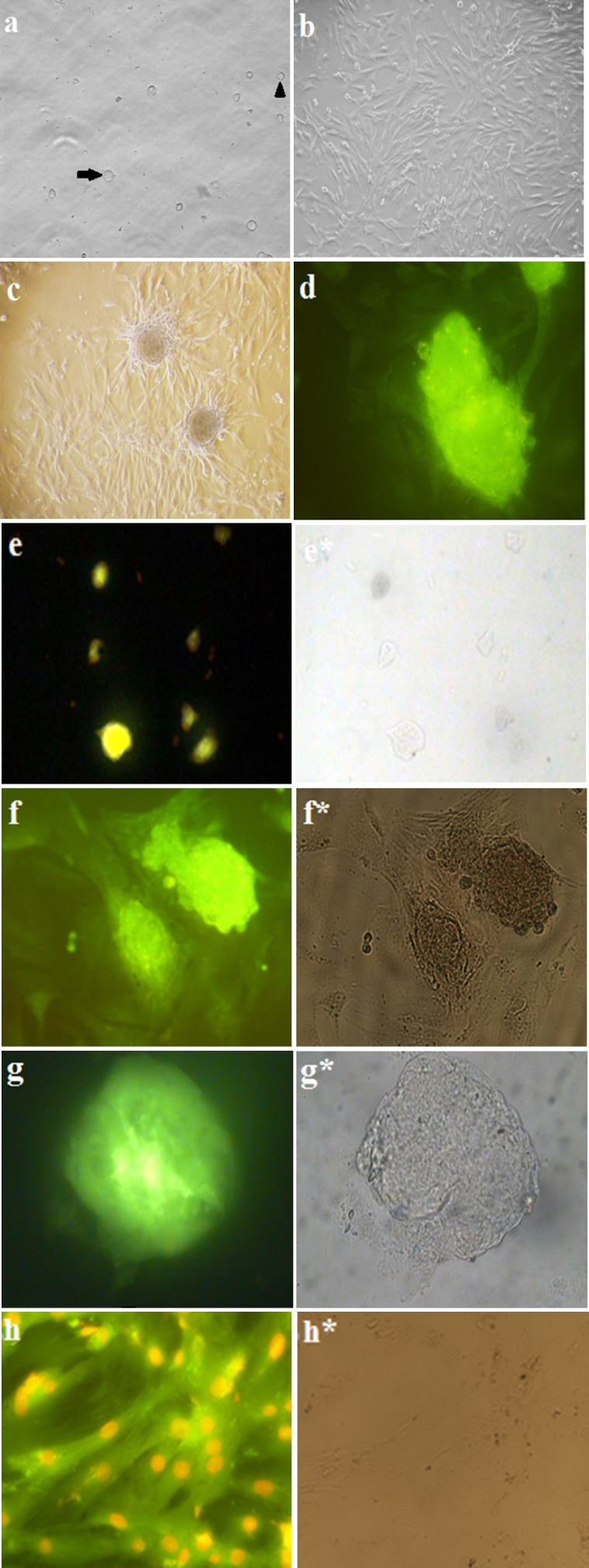
Isolation, culture and characterization of neonate mouse testicular cells. (a) Extracted cells from the seminiferous tubules after two steps of enzymatic digestion containing two cell types. The first (arrow tip), Sertoli cell, with granular feature and irregular outline and the second (arrow), SSCs, was round with a regular outline. (b) Sertoli cells proliferated and formed a monolayer of feeder layer cells. (c) SSCs colonies after two weeks of culture on the top of the Sertoli feeder layer cells. (d) SSCs colony immunostained with anti-Oct-4 antibody. (e) Oct-4 antibody positive reaction in CCE mouse embryonic stem cell line as positive control. (f) anti-CDH1 and (g) anti-PLZF immunostaining for characterization of SSCs colonies. (h) Cytokeratin-expressing Sertoli cells were counterstained with ethidium bromide to reveal cell nuclei. *indicate the phase contrast picture of the same immunofluorescence one. Magnifications: = × 200 and e=400.

The identity of the testis*-*specific cell types was confirmed by morphology assay and immunocytochemical evaluation. Sertoli cells were marked with rough border and granular look and formed a monolayer layer of feeder cells. SSCs included round remarkable borders, round nuclei, different (two or three) extraordinarily located nucleoli and formed prominent colonies (Figure 3). The immunoassay of sertoli cells showed positive cytokeratin immunoreactivity. The results also confirmed the positive Oct-4 reaction of stem cells in colonies, and SSCs clusters immunostained against specific markers CDH1 and PLZF (Figure 3).

### Cell viability and proliferation assays

After second digestion step, the survival rate of extracted cells was 89.13. After 2 weeks of culture, the viability percent of the cells in treated groups were higher relative to the control group in a concentration-dependent manner. This increase was significant in all treated groups except 0.0875 mg/ml of FC leaf extract relative to the control group. The highest survival rate was seen in 0.71 mg/ml of FC leaf extract, which were significantly different from that of control, 0.0875 and 0.175 mg/ml groups. In 1.42 mg/ml concentration, a significant difference was observed with control and 0.0875 mg/ml groups (P ≤ 0.05).

The comparison between the proliferation rate of control and treated groups showed that the cell number of all treated groups have increased significantly relative to the control group in a dose-dependent style. The three higher doses of 0.35, 0.71 and 1.42 mg/ml, had also significant differences with 0.0875 and 0.175 mg/ml groups (P ≤ 0.05; Table 2).

**Table 2:** Comparison between the mean ± SD of the viability percent and cell number (×10^6^) of neonate mouse testicular cells in control and treated groups in the presence of Fc leaf extract (0.0875, 0.175, 0.35, 0.71 and 1.42 mg/ml) with the same primary number of cells after 2 weeks of culture.

	**Control**	**0.0875mg/ml**	**0.175 mg/ml**	**0.35 mg/ml**	**0.71 mg/ml**	**1.42 mg/ml**
**Viability (%)**	**87.18 ± 1.24**	**88.7 ± 0.16**	**89.13 ± 0.21**^a^	**90.09 ± 0.49** b	**90.91 ± 1.35** c	**90.4 ± 0.76** d
**Cell number (×10^6^)**	**1.37 ± 0.05**	**1.52 ± 0.04** e	**1.63 ± 0.03** f	**1.81 ± 0.06** g	**1.89 ± 0.06** g	**1.89 ± 0.06** g

a Significant difference with the viability percent of control and 0.71 mg/ml group.

b Significant difference with the viability percent of control.

c Significant difference with the viability percent of control, 0.0875 and 0.175 mg/ml groups.

d Significant difference with the viability percent of control and 0.0875 group.

e Significant difference with the cell number of other groups except 0.175 mg/ml group.

f Significant difference with the cell number of other groups except 0.0875 mg/ml group.

g Significant difference with the cell number of control, 0.0875 and 0.175 mg/ml groups (P ≤ 0.05).

### Colony assay

#### Colony number

**Figure 4:**
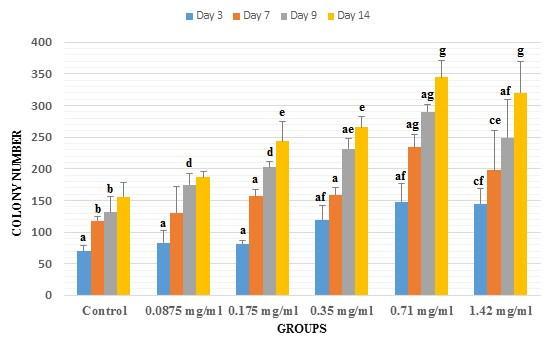
The mean ± SD of colony number was compared between control and experimental (0.0875, 0.175, 0.35, 0.71 and 1.42 mg/ml of common fig leaf aqueous extract) groups. (a) significant difference with other days in the same group. (b) significant difference with days 3 and 14 in the same group. (c) significant difference with days 9 and 14 in the same group. (d) significant difference with control in the same day. (e) significant difference with control and 0.0875 mg/ml of extract in the same day. (f) significant difference with control, 0.0875 and 0.175 mg/ml of extract in the same day. (g) significant difference with control, 0.0875 and 0.175 and 0.35 mg/ml of extract in the same day.

The mean number of colonies was compared between different days in all groups (Figure 4). Increase in the colony number was observed in all of the groups in a day-dependent way. The following results were obtained from intragroup evaluation: in control group, a significant difference was observed between the third day and the increased number of colonies in other days. Also, a significant difference was observed between 7^th^ and 9^th^ days compared to three and 14^th^ days. In the presence of 0.0875 mg/ml concentration of FC leaf extract, significant increases in days 7, 9 and 14 were detected compared to day 3. In 0.175 mg/ml group, significant differences in days 3 and 7 were observed relative to other days. Treatment with 0.35 and 0.71 mg/ml concentrations of FC leaf extract caused an increase in the colony number in a day-dependent manner so that significant differences in days 3, 7 and 9 were identified compared to other days. The group containing 1.42 mg/ml concentration of FC leaf extract showed significant increased colony number in days 9 and 14 relative to days 3 and 7 and a significant difference was observed between day 9 and other days.

Assessment of the colony number between different groups in the same days showed a dose-dependent increase pattern with detailed subsequent findings: three days after being cultured, colony number increased significantly in groups treated with 0.35, 0.71 and 1.42 mg/ml relative to the control, 0.0875, and 0.175 mg/ml concentrations groups. After 7 days, the colony number was significantly increased in the group treated with 0.71 mg/ml compared to the control, 0.0875, 0.175 and 0.35 mg/ml concentrations groups and in 1.42 mg/ml concentration-treated groups compared to control and 0.0875 mg/ml concentration groups. In 9th days, significant surges was seen in the mean number of colonies between the groups containing 0.0875 and 0.175 mg/ml concentrations of FC leaf extract with a lower mean in the control group. The other significant increases in day 9 was related to 0.35 mg/ml relative to the control and 0.0875 mg/ml concentration groups, 0.71 mg/ml concentration compared to control, 0.0875, 0.175 and 0.35 mg/ml groups, and 1.42 mg/ml concentration with control, 0.0875, and 0.175 mg/ml concentrations groups. In fourteen days, colony number increased significantly in groups treated with 0.175 and 0.35 mg/ml concentrations relative to the control and 0.0875 mg/ml concentration groups and in 0.71 and 1.42 mg/ml groups compared to the control, 0.0875, 0.175 and 0.35 mg/ml groups (Figure 4).

#### Colony diameter

**Figure 5:**
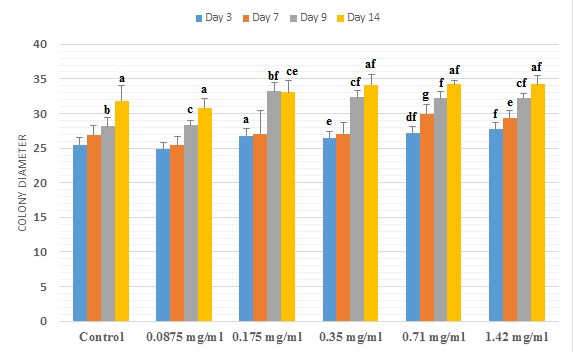
Comparison between the mean ± SD of diameter of colonies (µm) in control and experimental (0.0875, 0.175, 0.35, 0.71 and 1.42 mg/ml of common fig leaf aqueous extract) groups. (a) significant difference with other days in the same group. (b) significant difference with day 3 in the same group. (c) significant difference with days 3 and 7 in the same group. (d) significant difference with days 7 and 9 in the same group. (e) significant difference with 0.0875 mg/ml of extract in the same day. (f) significant difference with control and 0.0875 mg/ml of extract in the same day. (g) significant difference with control, 0.0875 and 0.175 and 0.35 mg/ml of extract in the same day.

As colony number, inter and intragroup evaluation for colony diameter was also performed (Figure 5). Increase in the colony diameter was observed in all of the groups in a day- and dose-dependent way. In intragroup assessment, the results were as the following: control group showed a significant increase in day 9 compared to day 3 and in day 14 relative to other days. In 0.0875 mg/ml concentration group, significant increases was observed in day 9 compared to days 3 and 7 and in day 14 relative to other days. In group receiving 0.175 mg/ml FC leaf extract, the lowest colony diameter in day 3 was significantly different from the other days. Increased colony diameter in days 9 and 14 was statistically different with day 3 and days 3 and 7, respectively. 0.35 mg/ml-treated group exhibited increased diameter in day 14, so that this increase was different from the other days. Moreover, increased diameter in day 9 relative to days 3 and 7 was significant. Treatment with 0.71 mg/ml concentration caused an increase in the colony diameter in a day-dependent manner so that the lowest colony diameter in day 3 was significantly different than days 7 and 9 and the highest one in day 14 was significantly different than the other days. Significant differences between day 9 with days 3 and 7 and between days 14 with other days was identified in the existence of 1.42 mg/ml FC leaf extract.

The following results were obtained from the evaluation of colony diameter between different groups in the same days: After 3 days, significant increases were observed in 0.35 mg/ml concentration group compared to 0.0875 mg/ml group and in groups containing 0.71 and 1.42 mg/ml concentrations compared to the control and 0.0875 mg/ml concentration groups. Significant increases in day 7 were related to 0.71 mg/ml relative to the control, 0.0875, 0.175 and 0.35 mg/ml concentration groups and 1.42 mg/ml concentration with 0.0875 mg/ml group. In day 9, colony diameter increased significantly in groups treated with 0.175, 0.35, 0.71 and 1.42 mg/ml concentrations relative to the control and 0.0875mg/ml concentration groups. Fourteen days after culture, colony diameter increased in 0.175 mg/ml treated group compared to 0.0875mg/ml concentration group. The other significant increases in this day were related to 0.35, 0.71 and 1.42 mg/ml concentrations compared to control and 0.0875mg/ml concentration groups (Figure 5).

### Gene expression analyses

**Figure 6:**
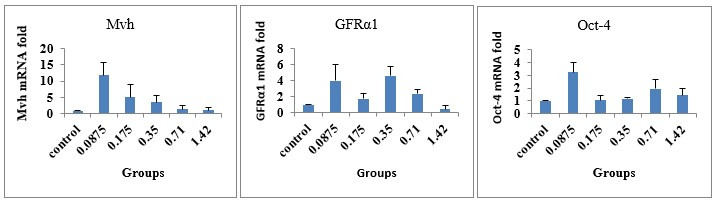
The mRNA fold expression (mean ± SEM) of Mvh, GFRá1, and Oct-4 genes relative to â-actin in control and experimental (0.0875, 0.175 and 0.35, 0.71 and 1.42 mg/ml of common fig leaf aqueous extract) groups. No significant differences were observed in the gene expression of control and experimental groups (P > 0.05).

To evaluate the expression of germ cell markers, the mRNA expression was analyzed with real time PCR assay of the following genes of Oct-4, Mvh, and GFRá1 in cultured testicular cells. In the current study`s analysis, the expression of Oct-4, Mvh, and GFRá1was observed in all of the groups. But, no significant differences were detected in the mentioned genes` expression of control and experimental groups (P > 0.05). The effect size for Oct-4, Mvh, and GFRá1 mRNA expression was 0.324, 0.373 and 0.418, respectively (Figure 6).

## Discussion

In this study, the *in vitro* effects of different doses of Fc leaf extract on testicular cells were evaluated using the assessment of viability percentage, proliferation rate and colony assay as the morphological criteria and molecular analyses. The mean of the survival and proliferation rates of cells in treated groups were significantly higher relative to the control group. This result suggested that Fc leaf extract not only had no any cytotoxic effects on SSCs, but also supported their maintenance.

Morena et al. (1996) reported that long-term culture of SSCs is difficult due to several reasons (Morena et al., 1996). These cells declined in the first week of cultivation. One of the likely reasons for this decrease is apoptosis (Aponte et al., 2006). In this procedure, reactive oxygen species (ROS) as essential intermediate messenger molecules, can be used by cells for signaling cascades` initiation in order to motivate caspases and endonucleases for cell killing (Naskar et al., 2010). Apoptosis can be prevented by antioxidants that counteract the free radicals and inhibit their bouts to the cells (Naskar et al., 2010). Culture cells are more sensitive to damages by ROS because of the higher *in vitro* free oxygen radicals produced by external factors such as light and oxygen in culture, cellular metabolism, and manipulations of cells in culture (Luvoni et al., 1996). So, it is rational to propose that the presence of antioxidants in the herbs might decrease cell apoptosis in the culture system. Leaves of Fc contain antioxidants as phenolic compounds that can prevent the oxidant-induced apoptosis (Gond and Khadabadi, 2008; Oliveira et al., 2009; Aghel et al., 2011). It is also described that caffeoylmalic acid (CMA), the most plentiful polyphenol in FC leaf, has antioxidant activity similar to that of catechin or vitamin C (Takahashi et al., 2014).

Another finding of this research was the similar morphology of colonies in all groups like to the clusters of earlier studies (Nagano et al., 2003; Boyer et al., 2012; Mohamadi et al., 2012). Colony assay is a way for the confirmation of SSC presence in culture and quantifying their activity (Boyer et al., 2012). Also, the size of clusters in each groups were diverse indicating that SSCs were not populated at the same time (Nagano et al., 2003; Mohamadi et al., 2012).

Based on this study`s results, the colony number and diameter increased during the culture period in all of the groups. This increasing pattern may be due to cell passage process during the culture which can prevent from the cell proliferation reduction caused by the unfortunate nutrition state, inadequate space and waste making of cells. This result was in agreement with Richards` findings which suggested that cell passage can stimulate cell proliferation by enhancing cell retrieval (Richards et al., 1999). In 2012, Mirzapour et al. (2012) reported that the number of human SSCs increased following passage procedure. Another likely description for this increasing pattern might be due to the existence of antioxidants in FC leaf, leading to the enhancement of viability and propagation of cultured SSCs in the presence of ROS during *in vitro* culture. Previous culture experiment demonstrated the proliferation of SSCs in the presence of estrogen (Miura et al., 2003). FC leaves were found to have phytoconstituents as flavonoids with estrogen*-*like action (Miksicek, 1995; Tamir et al., 2000; Fang et al., 2001; Vaya and Tamir, 2004). This property can be attributed to some examples of flavonoids such as Biochanin-A which is a phytoestrogen whose existence had been shown in the leaves of FC (Vaya and Mahmood, 2006; Vallejo et al., 2012). Phytoestrogens are compounds found in plants that have a similar structure and function of estrogen (Price and Rhodes, 1997). Also, the improvement of SSCs` proliferation in the presence of FC leaf extract may be due to FC containing saponin that act as testosterone like action (Haredy et al., 2017). In addition to estrogen, Tajik et al. (2012) reported the involvement of testosterone in SSCs enrichment in the culture. The leaves of Fc have been reported as an important source of vitamin C and E (Ghazi et al., 2012). The effect of vitamin C on the growth of caprine SSCs *in vitro* have been reported previously. The antioxidant activity of Vitamin c through decreasing ROS generation, have been assumed as the major contributing factor for the promotion of caprine SSCs proliferation *in vitro* in a dose-dependent way (Wang et al., 2014). Also, the key role of vitamins A, C, and E in support of rat undifferentiated type A spermatogonial proliferation have been shown by Boitani (Boitani et al., 1993).

Results of this study also showed that viability and proliferation rates of SSCs have increased in a dose-dependent style in the culture media. Moreover, colony morphology analysis of SSCs has revealed the surprisingly increased *in vitro* rates of colony number and diameter in the same way as the comparison between control and experimental groups showed higher number and diameter of colonies in two higher doses, 0.71 and 1.42 mg/ml concentrations of common fig leaf aqueous extract, separately for each time point relative to other groups. It seems that different effects on SSCs behavior can be the result from different concentrations of FC leaf extract. Dose-dependent activity of FC leaf extract have been reported by previous studies (Asadi et al., 2006; Krishna Mohan et al., 2007; Gond and Khadabadi, 2008; Oliveira et al., 2009; Amol et al., 2010; Lee and Cha, 2010; Aghel et al., 2011; Liu et al., 2011; Al Askari et al., 2013; Mahmoudi et al., 2016; Boukhalfa et al., 2018). Antioxidant is an important component in the fig leaf, and plays an important anti-apoptotic role (Gond and Khadabadi, 2008; Oliveira et al., 2009; Aghel et al., 2011). It has been demonstrated that the property of free radical scavenging of Fc leaf extract at different concentrations can appear in a dose-related fashion (Oliveira et al., 2009; Mahmoudi et al., 2016). Surveys such as that conducted by Asadi have shown the changes of lipid parameters in chicken liver in response to Fc leaf extract in a dose-dependent manner (Asadi et al., 2006). The strong and dose-dependent antioxidant activity and hypolipidemic effect on lipid parameters in triton-induced hyperlipidemic mice also has been reported by Boukhalfa (Boukhalfa et al., 2018). In this line, it has been recommended that the usage of FC leaf extract can protect CCl_4_ induced hepatic injury in a concentration-dependent way (Krishna Mohan et al., 2007; Aghel et al., 2011). Anti-inflammatory property of Fc leaf extract on carrageenan-induced rat in a concentration-dependent way was also reported (Amol et al., 2010). In addition to above mentioned effects, the extract of Fc leaves have an antimicrobial (Lee and Cha, 2010; Al Askari et al., 2013) and antiparasitic activities (Liu et al., 2011) which are dose-dependent.

In addition to the colony assay for the confirmation of germ stem cell, molecular evaluation was done to approve the nature of spermatogonia. Stemness (Oct-4), germ cell (Mvh) and germ stem cell (GFRá1) markers were analyzed after 2 weeks in the testicular cells cultured with or without FC leaf extract. Expression of Oct-4, Mvh and GFRá1 in all our groups were in consistent with the findings of earlier studies (Mirzapour et al., 2012; Mohamadi et al., 2012; Eslahi et al., 2013).

Oct-4, a transcription factor of the POU family, was used as an indicator of stemness (Nagano et al., 1998; Hofmann et al., 2005), which was also essential for the renewal of SSCs (Pesce et al., 1998). Observation of Oct-4 mRNA expression in all groups approved the stemness state of SSCs clusters. The results were in agreement with previous researches (Oke and Suarez-Quian, 1993; Mori et al., 1997; Pesce et al., 1998; Jeong et al., 2003; He et al., 2007; Absalan et al., 2008; Mirzapour et al., 2012; Mohamadi et al., 2012; Eslahi et al., 2013).

Mvh is an ATP-dependent RNA helicase which is silent in the somatic lineages and is expressed in pre and postmeiotic germ cells (Toyooka et al., 2003; Lee et al., 2006). Our results showed Mvh expression in the colonies of all groups; the same results have been also described in the former studies (Mirzapour et al., 2012; Eslahi et al., 2013).

Previous studies have reported that GFRá1 is a recognized specific marker of spermatogonia in numerous species (Dettin et al., 2003; Fujita et al., 2005; Eslahi et al., 2013) and have been identified as an indicator of SSCs rodents (Kolasa et al., 2012). The results of the present research showed the GFRá1expression in all groups. These findings were consistent with those of Mahaldashtian, who found that GFRá1was expressed in SSCs colonies.

The increased number and diameter of SSCs clusters with higher doses of extract, and the expression of stemness, germ cell and SSCs markers in all groups could indicate the efficacy of FC leaf extract in male germ stem cell proliferation. In line with our findings, several additional studies also described that spermatogonial clusters can proliferate in optimal culture conditions during 2 weeks of culture period and differentiated afterwards due to several reasons (Anjamrooz et al., 2006; Koruji et al., 2009, 2012; Mohamadi et al., 2012; Eslahi et al., 2013). However, further experimental investigations are needed to investigate the differentiation markers of SSCs.

## Conclusion

Based on the molecular findings, it seems that in the presence of FC leaf extract SSCs can proliferate and preserve their stemness state. Results of this study also showed that viability, proliferation, colony number and diameter of SSCs have increased in a dose-dependent manner in the culture media as the comparison between control and experimental groups indicated higher diameter and number of colonies in 0.71 mg/ml concentration of common fig leaf aqueous extract. There are plenty of studies that have shown *in vitro* SSCs proliferation in the short term culture period. The long-term proliferative property of spermatogonial stem cells with FC leaf extract was suitable for this cultural system regarding the proliferation of germ stem cells.

## Abbreviations

SSCs: spermatogonial stem cells; DMEM: Dulbecco’s modified eagle’s medium; ROS: Reactive oxygen species; Fc: *Ficus carica*; NMRI: National Medical Research Institute; FBS: Fetal bovine serum; GFRá1: GDNF family receptor alpha-1; CMA: Caffeoylmalic acid; ð_p_^2^: Partial Eta Squared; Mvh: Mouse vasa homolog; Oct-4: Octamer-binding transcription factor 4.
